# Global patterns of freshwater species diversity, threat and endemism

**DOI:** 10.1111/geb.12096

**Published:** 2013-07-03

**Authors:** Ben Collen, Felix Whitton, Ellie E Dyer, Jonathan E M Baillie, Neil Cumberlidge, William R T Darwall, Caroline Pollock, Nadia I Richman, Anne-Marie Soulsby, Monika Böhm

**Affiliations:** 1 Institute of Zoology, Zoological Society of LondonRegent's Park, London, NW1 4RY, UK; 2 Synchronicity Earth32a Thurloe Place, London, SW7 2HQ, UK; 3 Department of Genetics, Evolution and Environment, University College LondonGower Street, London, WC1E 6BT, UK; 4 Conservation Programmes, Zoological Society of LondonRegent's Park, London, NW1 4RY, UK; 5 Department of Biology, Northern Michigan UniversityMarquette, MI, 49855, USA; 6 Global Species Programme, IUCN219c Huntingdon Road, Cambridge, CB3 ODL, UK

**Keywords:** Congruence, conservation planning, decapods, diversity metric, geographical range, species richness

## Abstract

**Aim:**

Global-scale studies are required to identify broad-scale patterns in the distributions of species, to evaluate the processes that determine diversity and to determine how similar or different these patterns and processes are among different groups of freshwater species. Broad-scale patterns of spatial variation in species distribution are central to many fundamental questions in macroecology and conservation biology. We aimed to evaluate how congruent three commonly used metrics of diversity were among taxa for six groups of freshwater species.

**Location:**

Global.

**Methods:**

We compiled geographical range data on 7083 freshwater species of mammals, amphibians, reptiles, fishes, crabs and crayfish to evaluate how species richness, richness of threatened species and endemism are distributed across freshwater ecosystems. We evaluated how congruent these measures of diversity were among taxa at a global level for a grid cell size of just under 1°.

**Results:**

We showed that although the risk of extinction faced by freshwater decapods is quite similar to that of freshwater vertebrates, there is a distinct lack of spatial congruence in geographical range between different taxonomic groups at this spatial scale, and a lack of congruence among three commonly used metrics of biodiversity. The risk of extinction for freshwater species was consistently higher than for their terrestrial counterparts.

**Main conclusions:**

We demonstrate that broad-scale patterns of species richness, threatened-species richness and endemism lack congruence among the six freshwater taxonomic groups examined. Invertebrate species are seldom taken into account in conservation planning. Our study suggests that both the metric of biodiversity and the identity of the taxa on which conservation decisions are based require careful consideration. As geographical range information becomes available for further sets of species, further testing will be warranted into the extent to which geographical variation in the richness of these six freshwater groups reflects broader patterns of biodiversity in fresh water.

## Introduction

Freshwater ecosystems harbour a rich diversity of species and habitats. Their comparatively small distribution over the world's surface (less than 1%; Gleick, 1998) belies the far-reaching impact of the services that they provide. Although still incompletely surveyed, the current conservative estimate is that freshwater ecosystems provide suitable habitat for at least 126,000 plant and animal species (Balian *et al*., 2008). These species combine to provide a wide range of critical services for humans, such as flood protection, food, water filtration and carbon sequestration. Macroecological evaluations of understudied freshwater biota have been hampered by concerns over the generality of findings, due to restricted taxonomic representation. There have been notable studies of biotic diversity at a regional scale (e.g. Heino *et al*., 2002; Pearson & Boyero, 2009) and at other taxonomic levels (e.g. genera; Vinson & Hawkins, 2003), but global-scale analyses that synthesize information across taxonomic groups remain limited in number. Meanwhile, there is growing evidence that species in freshwater systems are under threat and in decline (e.g. Collen *et al*., 2009a; Galewski *et al*., 2011; Darwall *et al*., 2011a). The high level of connectivity of freshwater systems means that fragmentation can have profound effects (Revenga *et al*., 2005) and threats such as pollution, invasive species and disease are easily transported across watersheds (Dudgeon *et al*., 2006; Darwall *et al*., 2009). This lends urgency to the study of diversity and of the relative risk of extinction of species in freshwater ecosystems.

Highly biodiverse freshwater ecosystems are at risk from multiple interacting stresses that are primarily concentrated in areas of intense agriculture, industry or domestic activity. Water extraction, the introduction of exotic species, alteration of flow through the construction of dams and reservoirs, channelization, overexploitation and increasing levels of organic and inorganic pollution have added further stresses to freshwater ecosystems (Strayer & Dudgeon, 2010; Vörösmarty *et al*., 2010). In addition to these direct threats, climate change represents a growing challenge to the integrity and function of freshwater systems (Dudgeon *et al*., 2006). Nonetheless, a comprehensive assessment of freshwater species has yet to establish a full ecosystem-wide understanding of the distribution of freshwater species and the threats they face. The accomplishment of this goal is important, as it lays the foundation from which proactive conservation planning and conservation action can take place, as well as providing the baseline from which macroecological patterns of diversity, biotic change and ecological processes can be investigated and tested.

To date, much of our knowledge of broad-scale patterns of species distribution in freshwater systems, and the ecological processes that lead to them, has come from restricted subsets of species or small-scale data sets. There has been little synthetic work carried out at the global scale from which to form broad conclusions about patterns of diversity, endemicity and threats for freshwater species, although there are notable regional exceptions (e.g. Groombridge & Jenkins, 1998; Abell *et al*., 2008; Pearson & Boyero, 2009; Darwall *et al*., 2011a). Large-scale patterns of spatial variation in richness and endemism, and in the ecological attributes that dictate them – notably geographical range size – are central to many fundamental questions in macroecology and conservation biology (Orme *et al*., 2006). These include such issues as the origin of diversity, the potential impacts of environmental change on current patterns of richness and the prioritization of areas for conservation.

An understanding of the congruence of different metrics of biodiversity among taxa is an important first step in understanding the distribution of species in freshwater systems. Further, given that financial resources for conservation are limited, effective methods to identify priority areas for conservation to achieve the greatest impacts are crucial (Holland *et al*., 2012). A global perspective for the conservation of freshwater species has been largely constrained by a general lack of broad-scale information, leaving little option other than to use terrestrial centres of priority, which are likely to be unsuitable (Darwall *et al*., 2011b). The extent to which existing terrestrial protected areas protect freshwater species is unknown, but they are likely to be insufficient, as terrestrial protected areas rarely encompass the conservation of headwaters, are seldom catchment-based designs and do not consider the allocation of water downstream for biodiversity (Dudgeon *et al*., 2006; Darwall *et al*., 2009).

In this study, we evaluate a new global-level data set on the status of freshwater species derived from the sampled approach to IUCN red-listing (see Methods; Baillie *et al*., 2008; Collen & Baillie, 2010) and the global IUCN Red List database (IUCN, 2012). We evaluate the distribution of species richness and threat among freshwater species, identify centres of freshwater endemism and, using a heuristic approach, highlight key gaps in determining how freshwater conservation actions can be targeted at the most pressing cases.

## Materials and Methods

### Species data

Conservation assessments for species were generated according to the IUCN Red List Categories and Criteria (IUCN Species Survival Commission, 2012). The red-listing process has been extensively described in other articles (e.g. Mace *et al*., 2008; Hoffmann *et al*., 2010); briefly, an international network of freshwater species specialists were given the task of reviewing species-level data on taxonomy, measures of species distribution, population abundance trends, rates of decline, geographical range information and fragmentation in order to assign each species a Red List category. Each assessment was then reviewed by independent experts. The resulting assessments place each species in one of the following categories of extinction risk: extinct (EX); extinct in the wild (EW); critically endangered (CR); endangered (EN); vulnerable (VU); near threatened (NT); least concern (LC); and data deficient (DD). Data on broad habitat type (lakes, flowing water or marshes) and threat drivers (Salafsky *et al*., 2008) were collated for each species during the assessment process.

This resulted in a data set of 7083 freshwater species in six groups: mammals (*n* = 490; Schipper *et al*., 2008), reptiles (*n* = 57; Böhm *et al*., 2013), amphibians (*n* = 4147; Stuart *et al*., 2004), fishes (*n* = 630; IUCN, 2012), crabs (*n* = 1191; Cumberlidge *et al*., 2009) and crayfish (*n* = 568; N. I. Richman, Zoological Society of London, pers. comm.). Although a random representative sample of odonates (dragonflies and damselflies) has been assessed, this group was excluded from our analysis because distribution maps have not yet been completed. The freshwater reptile and fish assessments used in this analysis were selected and assessed for the sampled approach to red-listing, and therefore correspond to a representative random sample of species from these classes rather than assessments for all species in the group (Baillie *et al*., 2008; Collen & Baillie, 2010). Briefly, a sample of species was selected at random for mapping and risk assessment from a stable species list of the group; the sample size was sufficient to represent the level of threat faced by the group in question and the spatial distribution of the species (Baillie *et al*., 2008; see Supporting Information). The consequence of this is that cell richness values (see Analyses) must be compared on relative terms rather than absolute species number. All currently described species of freshwater crabs, mammals, crayfish and amphibians were included in this analysis. All of the species in this study are included in the IUCN Red List of Threatened Species online database (IUCN, 2012).

### Geographical data

The insular nature of freshwater habitats has led to the evolution of many species with small geographical ranges, which often encompass a single lake or drainage basin (e.g. Rossiter & Kawanabe, 2000; Dudgeon *et al*., 2006). Conservation in freshwater ecosystems must consider all activities in a catchment due to the high level of interconnectivity. It is therefore generally accepted that the river/lake basin or catchment is the most appropriate management unit for freshwater systems (Darwall *et al*., 2009). All species were mapped according to the IUCN schema (see Hoffmann *et al*., 2010), and all maps were created using ArcView/Map GIS software. For comparisons between species groups, range maps were projected onto a hexagonal grid of the world, resulting in a geodesic discrete global grid defined on an icosahedron and projected onto the sphere using the inverse icosahedral Snyder equal-area projection. This resulted in a hexagonal grid composed of cells with the same shape and area (7774 km^2^) across the globe. Distribution maps were used to assign each species to a biogeographical realm. Country occurrence was extracted from the IUCN data set to determine country endemism (defined as species confined to a geopolitical country unit; Ceballos & Ehrlich, 2002).

There are differences in sampling effort across species groups and geographical regions, such as between the well-studied Palaearctic mammals and the under-studied freshwater crabs of the tropical forests of Central Africa, but this compendium of data remains the best available source for our analyses. Congruence is likely to be adequate for broad-scale pattern identification using grid cells of around 1° (McInnes *et al*., 2009) and larger (Hurlbert & Jetz, 2007); our scale of analysis was a slightly less than 1°.

### Analyses

Some of the species in this analysis come from comprehensively assessed groups, with varying numbers of species, and some from groups in which a representative sample of the group was assessed. We therefore calculated a normalized richness score in order to make the groups comparable, and so that individual cell richness values were not dominated by the most numerous comprehensively assessed group(s). For each group, we calculated per cell species richness relative to the richest cell for that group in order to derive a synthetic pattern of mean diversity ranging from zero to one, with one representing the cell with highest species richness for that group, and zero representing cells with no species present. Thus, for a group with a highest species richness value of 100, a cell with 50 species would be normalized to 0.5, 40 to 0.4, and so on. We then calculated normalized global richness patterns by averaging threatened species (those species classified as CR, EN or VU), restricted-range species (defined as species with geographical ranges in the lower quartile of a taxon) and DD species across groups for all species.

To assess the extent to which taxonomic groups in this study show spatial congruence to one another, we generated spatial overlays of two measures of diversity – species richness and threatened-species richness – for each taxonomic group. Following studies that have evaluated similar patterns (e.g. Grenyer *et al*., 2006), we identified the richest 5% of grid cells for each taxon for both metrics of diversity. We also evaluated the distribution of species classified as DD in order to evaluate areas where gaps in our knowledge are aggregated. Amphibians are the most numerous freshwater group on the IUCN Red List, and the one with the longest history of investment in the red-listing process (Stuart *et al*., 2004). In order to evaluate whether amphibian distribution is reflective of that of other freshwater taxa, we calculated Pearson's correlations to evaluate pairwise comparisons between amphibians and all other taxonomic groups. Some cell locations are not inhabited by any organisms in this study. Such locations can inflate measures of covariation and association because their values for parameters of interest (in this case zero counts of species) are identical (the double zero problem; Legendre & Legendre, 1998); we therefore excluded these cells from our analyses. We accounted for the effects of spatial autocorrelation by implementing the method of Clifford *et al*. (1989), which estimates effective degrees of freedom based on spatial autocorrelation in the data and applies a correction to the significance of the observed correlation. We repeated this analysis using the richest 2.5 and 10% of cells, which made no qualitative difference to results (not reported).

We compared threat levels among taxa by habitat type using a binomial equality-of-proportions test. The true status of species classified as DD is unknown. In order to evaluate the uncertainty conferred by DD assessments on the proportion of threatened species, we calculated three measures of threat. These were: (1) a best estimate which assumes that DD species are threatened in the same proportion as those currently assessed in non-DD categories, [threatened/(assessed − EX − DD)]; (2) a minimum estimate or lower confidence limit that assumes DD species are not threatened, [threatened/(assessed − EX)]; and (3) a maximum estimate or upper confidence limit that assumes all DD species are threatened [(threatened + DD)/(assessed − EX)]. We generated confidence limits on these proportions using continuity correction as described by Newcombe (1998).

We calculated a correlation between gross domestic product (GDP; World Bank, 2011) and the number of country-endemic species, which we defined as those that are restricted to one country (Ceballos & Ehrlich, 2002), as a rudimentary estimation of how the resources available for conservation might relate to the need. We also ran the same analysis controlling for the size of each country (as larger countries are more likely to have greater numbers of endemic species). All statistical tests were carried out in R 2.12.1 (R Development Core Team, 2012), apart from the statistical analyses of congruence patterns, which were calculated using sam 4.0 (Rangel *et al*., 2010).

## Results

### Global freshwater species richness

**Figure 1:**
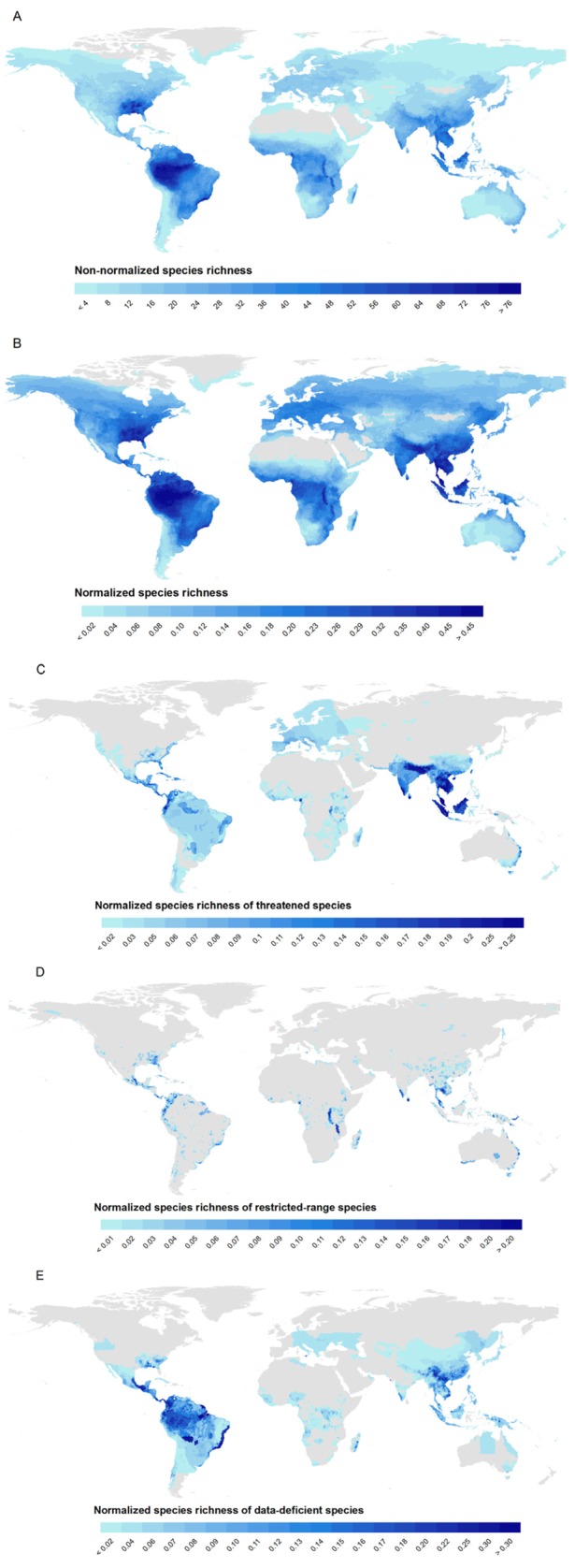
Global richness maps for freshwater species: (a) total non-normalized species richness; (b) total normalized species richness; (c) threatened species; (d) restricted-range species; and (e) data-deficient species.

Absolute freshwater diversity is highest in the Amazon Basin (Fig. 1a). Much of this pattern is driven by the high number of amphibians, which represent more than 50% of our data set. To account for this potential bias, we normalized richness from 0 to 1 across taxa (Fig. 1b), and we present both to highlight the differences. Doing so identifies several other important regions for freshwater diversity, specifically the south-eastern USA, West Africa across to the Rift Valley lakes, the Ganges and Mekong basins, and large parts of Malaysia and Indonesia. Brazil was the most diverse country, with over 12% of the total species count; the USA, Colombia and China each had 9–10%. Assemblages of threatened species show rather different general patterns of aggregation, with South and Southeast Asia by far the most threatened regions, with other notable centres of threat in Central America, parts of eastern Australia and the African Rift Valley (Fig. 1c, Table 1). Indo-Malaya had the greatest proportion of freshwater taxa, and the Palaearctic the lowest. Excluding the most species-rich group in our analysis (amphibians) had little discernible impact on the ranks (Table 1). Restricted-range species were patchily distributed across the tropics, with centres of endemism in the Rift Valley lakes (particularly Lake Malawi and Lake Tanganyika), Thailand, Sri Lanka and New Britain (Papua New Guinea) (Fig. 1d). The least-known area in terms of freshwater species diversity was in Central and South America, where the proportion of DD species was overwhelmingly highest (Fig. 1e; note that all but 69 of the 1758 DD species had sufficient location information to construct range maps).

**Table 1:** Total species richness and threatened-species richness for six groups of freshwater vertebrates and decapods, by biogeographical realm. Proportion threatened is best estimate (see Materials and Methods). Normalized proportion threatened gives an estimate for each group with equal weight, with rank order shown in the following column. The exclusion of amphibians reverses the rank of the two areas marked with an asterisk

	Total species	Threatened species	Proportion threatened	Normalized proportion threatened	Rank
Afrotropics	1174	263	0.27	0.19	5
Australasia	579	135	0.28	0.21	4*
Indo-Malaya	1796	422	0.37	0.28	1
Nearctic	759	140	0.20	0.23	2
Neotropics	2506	654	0.35	0.22	3*
Oceania	11	0	0.00	0.00	7
Palaearctic	695	142	0.23	0.18	6

Table 2 shows that many countries with high freshwater diversity – so-called ‘megadiverse’ nations – also exhibited a high degree of country or ‘political’ endemism (Ceballos & Ehrlich, 2002). In our data set, 62% of the species were found to be ‘politically endemic’ and only 12% had ranges which span five or more countries. Megadiverse nations with more than 50% endemism of freshwater species included Madagascar (96%), Australia (84%), the USA (73%), Mexico (59%), China (55%) and Brazil (51%). The USA had the highest absolute political endemism, with almost 500 endemic freshwater species. The correlation between GDP and number of politically endemic species is strongly and significantly positive (*r* = 0.78, *P* < 0.001, d.f. = 22).

**Table 2:** Richness of freshwater vertebrate and decapod species by country, ranked by proportion of endemic species. Area-adjusted rank shows how the rank order of countries changes when the size of each country is taken into account

Country	Area (km^2^)	Number of species	Number of endemic species	Proportion endemic	Area-adjusted rank
Tanzania	945,087	189	181	0.96	8
China	9,706,961	388	325	0.84	18
Argentina	2,780,400	681	496	0.73	9
Guyana	214,969	361	214	0.59	1
Bolivia	1,098,581	643	351	0.55	5
Angola	1,246,700	861	436	0.51	4
DR Congo	2,344,858	368	162	0.44	13
Australia	7,692,024	673	269	0.40	17
Brazil	8,514,877	420	151	0.36	24
Colombia	1,141,748	372	117	0.31	11
India	3,166,414	331	88	0.27	20
Lao PDR	236,800	325	88	0.27	2
Cameroon	475,442	394	103	0.26	7
Ecuador	256,369	368	90	0.24	3
Malaysia	330,803	256	53	0.21	10
Peru	1,285,216	233	50	0.21	16
Indonesia	1,904,569	329	62	0.19	19
Myanmar	676,578	241	42	0.17	14
Mexico	1,964,375	249	40	0.16	23
Vietnam	331,212	165	25	0.15	12
Venezuela	912,050	167	19	0.11	22
Panama	75,417	237	23	0.10	6
Madagascar	587,041	279	24	0.09	15
Thailand	513,120	189	13	0.07	21
USA	9,629,091	174	10	0.06	25

### Distribution of risk among taxa and habitat

**Figure 2:**
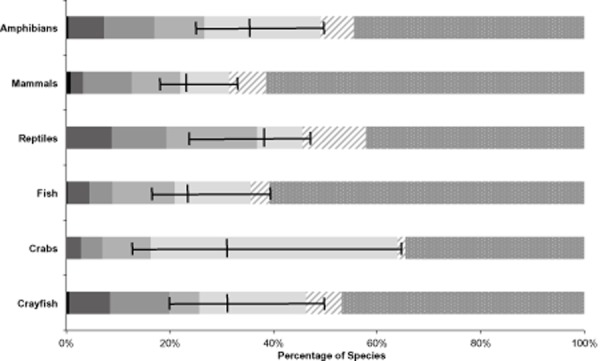
Extinction risk of global freshwater fauna by taxonomic group. Central vertical lines represent the best estimate of the proportion of species threatened with extinction, with whiskers showing confidence limits. Data for fish and reptiles are samples from the respective group; all other data are comprehensive assessments of all species (*n* = 568 crayfish, 1191 crabs, 630 fish, 57 reptiles, 490 mammals and 4147 amphibians). Solid colours are threatened species, from left to right: black, extinct; darkest grey, critically endangered; mid-grey, endangered; light grey, vulnerable; lightest grey, data deficient. Patterned bars are non-threatened species: hatched, near threatened; dotted, least concern.

Almost one in three freshwater species is threatened with extinction world-wide [proportion threatened 0.32; 95% confidence interval (95% CI) 0.24–0.49] (Fig. 2). All groups evaluated in this analysis exhibit a higher risk of extinction than their terrestrial counterparts (proportion of terrestrial species threatened 0.24; 95% CI 0.21–0.32; data from Collen *et al*., 2009b). There is remarkably little geographical variation in the threat to freshwater species at the level of geographical realms, with the proportion of threatened freshwater taxa ranging between 0.23 and 0.36, excluding Oceania (Table 1). Reptiles are potentially the most threatened freshwater taxa, with nearly half of species threatened or near threatened (Fig. 2). There is stark variation between groups, but with no discernible consistent pattern separating vertebrates from decapods (Fig. 2). Levels of data deficiency are much higher in freshwater crabs, leading to greater uncertainty over threatened status. The proportions of threatened and DD crayfish are similar to those of amphibians.

**Figure 3:**
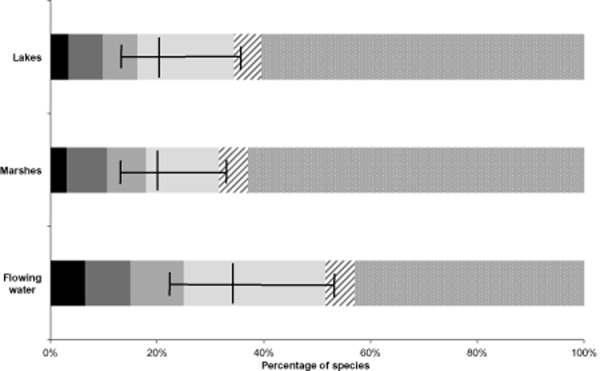
Global threat levels for three freshwater habitats. Central vertical lines represent the best estimate of the proportion of vertebrate and decapod species threatened with extinction, with whiskers showing confidence limits. Numbers of species are 2797 in lakes, 1281 in marshes and 5374 in flowing water. Solid colours are threatened species, from left to right: black, extinct; darkest grey, critically endangered; mid-grey, endangered; light grey, vulnerable; lightest grey, data deficient. Patterned bars are non-threatened species: hatched, near threatened; dotted, least concern.

Freshwater vertebrates have a very similar extinction risk to decapods in freshwater ecosystems (proportion of vertebrates threatened 0.318, 95% CI 0.25–0.46; proportion of decapods threatened 0.315, 95% CI 0.19–0.58). Less detailed knowledge of invertebrate biology and threat led to slightly wider confidence limits around estimated threat levels (due to greater proportion of DD classifications). The type of freshwater habitat also appeared to be important in determining threat levels (Fig. 3), with 34% of species inhabiting lotic habitats being under threat (rivers and streams; proportion threatened 0.34, 95% CI 0.53–0.24) compared with 20% of marsh species (proportion threatened 0.20, 95% CI 0.34–0.15) and lake species (proportion threatened 0.20, 95% CI 0.36–0.15).

### Cross-taxon congruence

**Figure 4:**
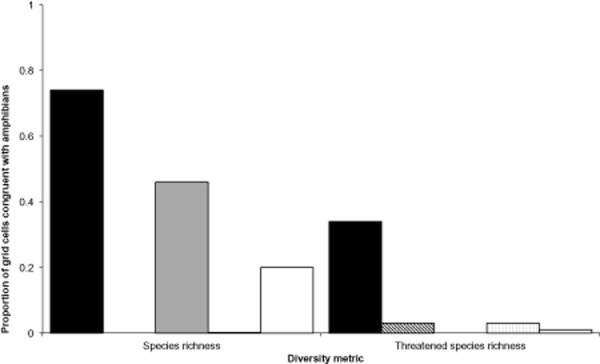
Cross-taxon congruence for two metrics of diversity, species richness and threatened-species richness. Bars show the proportion of freshwater ecosystems shared between five different freshwater taxa and amphibians: black bar, crabs; diagonal hatching, crayfish; grey, fish; vertical hatching, mammals; white, reptiles.

Pairwise analysis of geographical distribution between taxa showed that no single species group exhibited a consistent pattern of congruence with other taxa (Table 3). For example, the distributions of crabs and crayfish are largely exclusive, with little geographical overlap on a global scale. There were marked differences in the congruence of taxa under different metrics of diversity, with species richness and threatened-species richness showing rather different patterns. The greatest congruence of species richness was observed between amphibians and crabs (proportion of shared grid cells = 0.74). The congruence of threatened-species richness for these two groups was far lower (proportion of shared grid cells = 0.34). Crayfish showed the least congruence with other taxa, with a maximum congruence of 0.13 shared grid cells with reptiles and the lowest congruence with crabs. There were no significant correlations between amphibians and the other taxonomic groups when the richest 5% of cells were compared (Table 4, Fig. 4).

**Table 3:** Correlation matrix of spatial congruence between geographical ranges of freshwater vertebrate and decapod taxa world-wide. The proportion of grid cells for each pairwise comparison of taxa are given for two measures of diversity, (left) total species richness and (right) threatened-species richness. A value of 1 implies perfect correlation between taxa. The comparison is presented for the richest 5% of grid cells for each taxon for both metrics of diversity

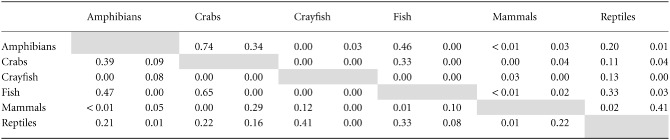

**Table 4:** Correlation with other groups of the richest 5% of non-zero cells for amphibians. Values of *F*, *P* and d.f. were corrected for spatial autocorrelation using the method of Clifford *et al*. (1989), here denoted ‘corr’

Group	*n*	Pearson's *r*	*F*	*F*_(corr)_	d.f.	d.f._(corr)_	*P*	*P*_(corr)_
Mammals	828	0.217	40.8	1.3	826	26.2	< 0.001	0.266
Reptiles	828	−0.058	2.8	0.1	826	32.4	0.095	0.743
Fish	828	−0.047	1.9	1.7	826	744.1	0.173	0.197
Crayfish	828	−0.042	1.5	0.4	826	241.9	0.222	0.509
Crabs	828	0.334	164.0	3.4	826	26.8	0.000	0.078

### Drivers of threat

**Figure 5:**
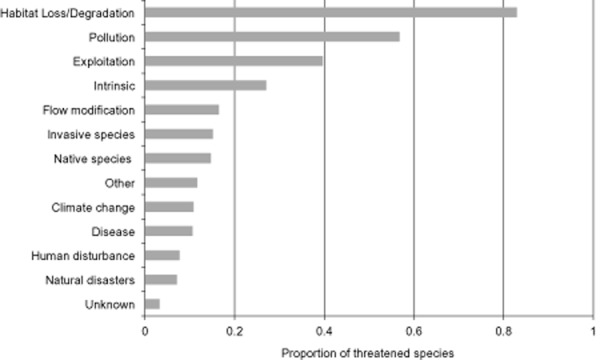
Global drivers of threats causing decline of freshwater vertebrate and decapod species (*n* = 1674 threatened species).

Three processes predominantly threatened freshwater species: habitat loss/degradation, water pollution and over-exploitation (Fig. 5). Of these, habitat loss/degradation was by far the most prevalent, affecting more than 80% of threatened species. The main proximate drivers of habitat loss and degradation were agriculture, urbanization, infrastructure development (particularly the building of dams) and logging. Any simplistic conclusions are complicated by the interactions between different threat processes (for example, water pollution can be caused by a variety of factors, including chemical run-off from intensive agriculture, sedimentation resulting from logged riparian habitat, and domestic waste water from urban expansion). The relative importance of threat drivers shows wide variation among the taxa studied: 98% of threatened crabs and 74% of threatened fish were at risk due to pollution. Overexploitation was a greater threat to crayfish and reptiles (71 and 86% of threatened species, respectively). Only half of threatened freshwater fish were affected by habitat loss, compared with 90% of mammals and amphibians and 96% of crabs.

## Discussion

Our study suggests that freshwater species across a range of vertebrate and decapod groups are consistently under a greater level of threat than those resident in terrestrial ecosystems (Collen *et al*., 2012). These patterns of threat are mediated by high rates of habitat loss and degradation, pollution and overexploitation, and are particularly problematic in species inhabiting flowing waters. Overall, congruence between the distributions of two metrics of diversity for the taxa in this study at this spatial resolution was low: no one group exhibits a consistent pattern of congruence with other taxa. The conservation status of vertebrate species may therefore not be an accurate indicator of the status of all the non-vertebrate freshwater taxa (as suspected globally by Dudgeon *et al*., 2006). This lack of congruence at the subcatchment resolution has also been demonstrated at a continental scale for African freshwater species (Darwall *et al*., 2011b), and at smaller scales in aquatic ecology (e.g. Heino *et al*., 2002, 2003). Our results therefore have important implications for understanding global patterns of both diversity and extinction risk. Foremost, because there are marked spatial patterns in the distribution of richness and extinction risk across the freshwater taxa for which we had information, this implies that not only are there areas of greater conservation concern, but also that those areas are likely to differ, at least at a broad scale, depending on the taxonomic groups being evaluated. Identifying the drivers both of freshwater diversity and of the traits that confer elevated risk of extinction are clear goals for macroecologists and those concerned with biotic impoverishment.

We were able to take the global distribution of species in six taxonomic groups into account in our analyses, including two broadly distributed freshwater decapod groups. One conclusion of our study must be that distributional information for other invertebrates remains sparse. As knowledge of the geographical ranges and relative risks of extinction in other freshwater taxa becomes available – notably freshwater molluscs, plants and odonates – it is feasible that this broad-scale pattern may change. Given the small ranges that many of these additional species are likely to exhibit, it seems unlikely that a much more congruent picture of shared centres of threat and richness will emerge. Our findings emphasize the need for a greater understanding of the status of freshwater biodiversity, and its distribution across the globe, particularly of important functional communities such as detritivores or shredders (e.g. Boyero *et al*., 2012).

Our analysis was made more complex by the need to integrate distribution data for sampled and comprehensively assessed groups in order to gain a global picture of richness and threat to freshwater species. Although simulations show that global diversity patterns for comprehensively known groups such as amphibians and mammals are consistently re-created with the random resampling of around 5–10% of species (B.C., unpublished data), our sample for freshwater fish lies at the lower end of this range, principally because the sample was drawn from among all fish (both marine and freshwater species; Baillie *et al*., 2008). Although the true regional-scale distribution patterns of freshwater fish will not be known until the comprehensive compilation of distributional data for that group has been achieved, we have some confidence that our sample is broadly representative at the scale of our analysis. Nevertheless, our approach is susceptible to omission errors, which could alter regional-scale patterns in particular. In cells where species are not sampled, relative richness values will be underestimated. This could be particularly the case for threatened species, which tend to have smaller ranges.

Across all groups, the more affluent countries – with a richer history of research on freshwater species – will be more comprehensively surveyed, which could in turn bias the results. Given the rate of discovery of new species in freshwater ecosystems (e.g. an average of one species of fish per day has been described over the past 20 years; Eschmeyer & Fong, 2012) it would be pertinent to understand where new species might come from and to account for their impact on diversity patterns (Collen *et al*., 2004; Diniz-Filho *et al*., 2005).

Given the apparent lack of congruence between both metrics of diversity that we tested (species richness and threatened-species richness), and between the six taxonomic groups that we were able to include in this study, our findings raise a macroecological question. Do the determinants of range differ among these freshwater groups, particularly among wide-ranging and restricted-range species? Comparatively little is known about the determinants of range size. This is particularly true for widespread species, although a global analysis of range size in amphibians revealed that temperature seasonality was the primary determinant (Whitton *et al*., 2012), and a regional analysis of Afrotropical birds suggested that range margins are concentrated in the most heterogeneous areas of habitat (McInnes *et al*., 2009). Macroclimatic variables may be range-limiting factors, but principally for wide-ranging species (Jetz & Rahbek, 2002; Rahbek *et al*., 2007; Tisseuil *et al*., 2013). Determinants of range are likely to be the product of refugia (from past extinctions or glacial maxima), or high rates of allopatric speciation (Jetz & Rahbek, 2002) for restricted-range endemic species. In freshwater systems, it is likely that the impermeability of the margins of catchments to less motile species will be the key driver of range margins (Tedesco *et al*., 2012). A landscape impermeability matrix may therefore act as a suitable surrogate for defining the range of additional taxa in freshwater ecosystems, particularly for those taxa whose range margins coincide with the geographical components that determine watersheds.

We found that the types of threats that are driving freshwater species into categories of high risk were similar among the six species groups that we tested, which suggests there are potential short-cuts for conservation organizations addressing those threats that could reap multiple benefits. Land-use change driving habitat loss and degradation affects the majority of threatened freshwater species. Success in addressing these ultimate drivers of loss lies in tackling the proximate threats (from agriculture, forestry and infrastructure development) using more sustainable production methods, along with underlying causes such as a lack of control of land-use planning in many highly biodiverse countries. Freshwater ecosystems are frequently affected by a multitude of threats, and status assessments across a range of metrics of biodiversity suggest that these are often of greater magnitude than those for terrestrial species (Revenga *et al*., 2005).

Undertaking to conserve the variety of threatened freshwater taxa identified here means spreading conservation efforts over wider regions. Regional-scale studies could provide the means to make astute and efficient decisions at the most relevant scale (e.g. Darwall *et al*., 2011a). Although our data set will not tell the full story of the relationship of endemic species due to the use of some sampled data sets, the fact that we found a strong positive correlation between number of country-endemic species and GDP could be both positive and negative for conservation of freshwater biodiversity. On one hand, it might mean that economically richer countries are more able to look after freshwater biodiversity, but conversely, there is a danger that these more affluent nations might be more likely to develop and degrade their freshwater ecosystems by having the capital to make wholesale changes. Most nations are signatories to the Convention on Biological Diversity, and are bound by the 20 Aichi Biodiversity Targets (Convention on Biological Diversity, 2010), at least three of which require metrics of their performance in protecting freshwater biodiversity. For example, Target 11 is to conserve 17% of inland water by 2020, Target 14 is to restore ecosystems providing essential services ‘including services related to water’, and Target 6 aims to ensure that ‘all fish and invertebrate stocks and aquatic plants are managed and harvested sustainably by 2020’ (Convention on Biological Diversity, 2010). Trends in extinction risk, abundance and geographical range of a wide variety of freshwater species will be integral to answering whether or not these commitments have been met.

One area of interest for freshwater macroecologists could be to establish the empirical links between the status of freshwater species and the functions that they provide to humans, particularly for common and abundant species in widespread decline. The links between freshwater biodiversity and human livelihoods appear to be much more direct than for other ecosystems (e.g. water filtration, nutrient cycling and the provision of fish and other protein). However, the extent to which such freshwater ecosystem services rely on high species diversity or other aspects of functional and trait diversity remains largely unknown (Cardinale *et al*., 2012). To help answer such questions in freshwater ecosystems, taxonomic groups such as molluscs should be high on the list for assessment on the IUCN Red List, specifically due to the ecosystem services that they provide.

Our study represents the largest compendium of geographical range data for freshwater species that we are aware of, and builds on bioregional studies such as Abell *et al*. (2008). It shows that multiple metrics of diversity across a range of taxa should be considered to answer broad-scale questions about freshwater species range dynamics and conservation status. However, we caution that the coverage amassed is far from complete, and efforts should be made to fill both taxonomic and geographical gaps in order to verify the patterns that we have identified. Our study highlights the type and degree of threat now facing freshwater species and so demonstrates the urgency for completing an assessment of freshwater diversity, possibly down to the scale of subcatchments, to inform on-the-ground conservation action to safeguard these species.

## Notes

BIOSKETCH The two research teams involved in this analysis are the Indicators and Assessments Unit at the Zoological Society of London (http://www.zsl.org/indicators) and the Freshwater Biodiversity Unit at IUCN (http://www.iucn.org/). Both have research aims concerning the understanding of global change in biodiversity. Author contributions: B.C., F.W. and M.B. conceived the ideas; E.E.D., F.W., N.C., W.R.T.D., C.P., N.I.R., J.E.M.B. and A.-M.S. collected the data; F.W., M.B. and B.C. analysed the data; B.C., F.W., W.R.T.D. and M.B. led the writing, to which all authors contributed.

## SUPPORTING INFORMATION

**Appendix S1** Reptile and fish species in our analyses of freshwater species. **Figure S1** Proportion of freshwater fish species by biogeographical realm.
