# Supplementary material for: Global patterns of freshwater species diversity, threat and endemism
Source: Glob Ecol Biogeogr. 2013 Jul 3;23(1):40–51. doi: 10.1111/geb.12096 (PMC4579866; doi:10.1111/geb.12096)
Supplement: Supplementary file 1 — Appendix S1 Reptile and fish species in our analyses of freshwater species. Figure S1 Proportion of freshwater fish species by biogeographical realm. [file geb0023-0040-sd1.docx]

*Global Ecology and Biogeography*

**Supporting Information**

**Global patterns of freshwater species diversity, threat and endemism**

Ben Collen, Felix Whitton, Ellie E. Dyer, Jonathan E. M. Baillie, Neil Cumberlidge, William R. T. Darwall, Caroline Pollock, Nadia I. Richman, Anne-Marie Soulsby and Monika Böhm

**Appendix S1** Reptile and fish species in our analyses of freshwater species are derived from a sample of the total number of species in the group (Baillie *et al.*, 2008; Collen *et al.*, 2009; Collen & Baillie, 2010). Figure S1 shows how the distribution of species by biogeographical realm varies between the total known distribution of freshwater fish species, and those repre­sented in the sample. The sample slightly over-represented Australasian species, and slightly underrepresented Nearctic species (*P* < 0.05, binomial equality of proportions test). All other tests were non-significant.

**Figure S1** Proportion of freshwater fish species by biogeographic realm. Black bars are the proportions of sampled species, grey bars are proportions of total reported number of species (Lévêque *et al.*, 2008). An asterisk (*) indicates significant difference (*P* > 0.05) in a binomi­al equality of proportions test between the pairs.

**References**

Baillie, J.E.M., Collen, B., Amin, R., Akcakaya, H.R., Butchart, S.H.M., Brummitt, N., Meagher, T.R., Ram, M., Hilton-Taylor, C. & Mace, G.M. (2008) Towards monitoring global biodiversity. *Conservation Letters*, **1**, 18–26.

Collen, B., Ram, M., Dewhurst, N., Clausnitzer, V., Kalkman, V., Cumberlidge, N. & Baillie, J.E.M. (2009). Broadening the coverage of biodiversity assessments. *Wildlife in a changing world: an analysis of the 2008 IUCN Red List of Threatened Species* (ed. by J.-C. Vié, C. Hilton-Taylor and S.N. Stuart), pp. 67–76. IUCN, Gland, Switzerland.

Collen, B. & Baillie, J.E.M. (2010) The barometer of life: sampling. *Science*, **329**, 140.

Lévêque, C., Oberdorff, T., Paugy, D., Stiassny, M.L.J. & Tedesco, P.A. (2008) Global diversity of fish (Pisces) in freshwater. *Hydrobiologia*, **595**, 545–567.
